# Vaccine use in Canadian cow-calf herds and opportunities for improvement

**DOI:** 10.3389/fvets.2023.1235942

**Published:** 2023-08-09

**Authors:** Madelana M. Lazurko, Nathan E.N. Erickson, John R. Campbell, Sheryl Gow, Cheryl L. Waldner

**Affiliations:** ^1^Department of Large Animal Clinical Sciences, Western College of Veterinary Medicine, University of Saskatchewan, Saskatoon, SK, Canada; ^2^Public Health Agency of Canada, Saskatoon, SK, Canada

**Keywords:** vaccine, cow-calf, beef cattle, disease control, management

## Abstract

Vaccinations are one of the most impactful tools available to cow-calf producers to control within herd disease and later, in feedlots. While vaccine use has been studied across Canada, inconsistent and variable regional data makes analysis and interpretation difficult. The objective of this study was to describe vaccination protocols and factors associated with vaccine use in Canadian cow-calf herds and define associations between vaccine use and productivity outcomes. Surveys describing vaccine use in 2020 were collected from 131 cow-calf herds (40 eastern, 91 western), recruited through a national beef cattle surveillance program. Ninety-two percent of cows and replacement heifers, and 72% of bulls were vaccinated with Infectious Bovine Rhinotracheitis (IBR), Bovine Viral Diarrhea Virus (BVDV), Parainfluenza3 Virus (PI3), and Bovine Respiratory Syncytial Virus (BRSV). At least half of cows and bulls were vaccinated for clostridial pathogens and cows and heifers for viral calf scours. Clostridial vaccines were significantly more likely to be used in western Canada compared to eastern Canada. While 92% of producers vaccinated suckling calves against IBR/BRSV/PI3, only 47% provided a second vaccine prior to weaning; 78% of calves were also vaccinated at least once for BVDV before weaning. Producers who vaccinated calves against IBR/BRSV/PI3 before 3 months of age provided a second dose prior to weaning more often than producers who administer the first IBR/BRSV/PI3 vaccine later. Vaccine use has increased across Canada, particularly in calves, prior to weaning. Relative to label recommendations for annual vaccination, clostridial vaccines were generally underutilized in cows and bulls, and by producers in eastern Canada as compared to western Canada. Opportunities also exist to improve adherence to label recommendations for the booster dose of scours vaccine when used in bred replacement heifers. Protocols including product choices, the timing and boosting of respiratory vaccines in nursing calves vary widely across herds. Use of intranasal vaccines in neonatal calves less than 2 weeks old has increased in western Canada compared to previous reports. There is a need to better understand how timing of vaccination in nursing calves contributes to effectiveness, for respiratory disease in nursing and weaned calves.

## 1. Introduction

Vaccination of beef cattle has proven effective in stimulating immune response and reducing disease burden across a range of study designs (1–8). However, other studies of vaccine use in cow-calf herds and at feedlot entry have shown more variable effectiveness (9–18). Adherence to evidence-based vaccine protocols can improve animal health and increase production efficiency through control and prevention of economically important diseases. For example, two recent meta-analyses summarized the evidence for mitigation of abortion risk and other negative reproductive consequences of Infectious Bovine Rhinotracheitis (IBR) and Bovine Viral Diarrhea Virus (BVDV) (2, 7).

Vaccinating against reproductive and clostridial diseases is commonly recommended by veterinary practitioners (19). The American Association of Bovine Practitioners (AABP) identified the following targets as core vaccines for all beef cattle: IBR, BVDV, Parainfluenza3 Virus (PI3), Bovine Respiratory Syncytial Virus (BRSV), and clostridial diseases with the label for all licensed products either requiring or recommending annual revaccination (20). The AABP recommends modified live viral (MLV) vaccines for IBR and BVDV due to the increased efficacy compared to killed or inactivated vaccines (20).

Other vaccines should be recommended by veterinarians on an individual herd basis, based on specific risk factors and geographical location (20). A number of studies have documented causes of morbidity and reasons for antimicrobial use in cow calf herds. For example, a 2015/2016 survey of Ontario cow-calf producers and a 2017 survey of western Canadian cow-calf producers found that neonatal calf scours (13–52%) and pre-weaning calf respiratory disease (15–16%) were important causes of total pre-weaning mortality (21, 22). Respiratory disease is the most reported reason for antimicrobial use in both cow-calf herds and feedlots (23, 24). Commercial vaccines labeled to aid in the control of scours and respiratory disease are available and are commonly used in cow-calf herds (25), although the results for published field trials are variable for both calf scours and respiratory disease preweaning (10, 12).

Timing of vaccine administration must be considered for optimizing effectiveness particularly for control of respiratory disease. While vaccination before weaning has been shown to reduce treatment for respiratory disease relative to vaccination at or after weaning and again at feedlot arrival (26, 27), improved performance across the entire feeding period was not observed in these studies for calves vaccinated before weaning. The evidence supporting beneficial effects of administration of respiratory vaccines at feedlot arrival is even more limited with many studies showing no or negative associations with vaccination (26, 28–31). The lack of current economic incentive for cow-calf producers to fully vaccinate calves prior to sale to prevent respiratory disease in feedlots limits participation by cow-calf producers.

Literature regarding national adoption and use of vaccines across the Canadian cow-calf industry is scarce. However, recent industry reports using cow-calf producer survey data have demonstrated regional vaccine use within Canada. Western Canadian studies have previously described vaccination protocols for the following years: 2016/2017 (21), 2010 (5) and 2001/2002 (6). Eastern Canadian cow-calf producers have been surveyed regarding vaccine use in Atlantic Provinces (2017) (32), Ontario (2015/2016) (33, 34), and northern Quebec (2015) (34).

A previous western Canadian surveys found that cow-calf producers used at least one vaccine in cows, heifers, unweaned calves, weaned calves and bulls in 97, 97, 96, 57 and 72% of herds, respectively (25). The most common vaccine targets for calves were clostridial diseases, and respiratory and reproductive vaccines were the most common vaccines for bulls, cows and replacement heifers (25). Producers from eastern Canada had previously reported vaccine use among cows, heifers, calves and bulls in Ontario of 70, 72, 88, and 59% and in northern Quebec of 72, 78, 94 and 68%, respectively (33, 34). Similar data were not reported for Atlantic herds, but 73% of producers reported vaccinating cattle, and 45% vaccinated females prior to breeding (32).

The primary objective of this study was to describe vaccine adoption in cow-calf herds across Canada and provide a better understanding of the types of vaccines used and timing of administration. Results will be used to investigate regional differences and opportunities for improvement in vaccination uptake and adherence to recommended protocols. The secondary objective of this study was to examine factors associated with vaccine use and explore potential associations between vaccine use and herd productivity outcomes.

## 2. Materials and methods

This study was approved by the University of Saskatchewan’s Behavioral Research Ethics Board (Beh-REB#309).

### 2.1. Survey design and content

A paper-based survey was developed based on a tool tested and used in western Canadian herds in 2016 (25). The survey requested herd data from January 1, 2020, to December 31, 2020, and was split into two parts: Part A inquired about herd characteristics, management practices, and technology adoption (data reported elsewhere), and Part B asked about specific herd vaccination protocols. Producers described vaccine use by completing a series of open text tables for each production group: bulls, cows, replacement heifers, weaned calves, and suckling calves. Each table was split into rows, based on vaccine target (e.g., bulls: reproductive, clostridial, respiratory, foot rot, anthrax, and other) with space to allow for multiple vaccines per target. Commercial vaccine names were recorded, and time of administration relative to other herd management activities was selected from a list (e.g., bulls: before breeding, after breeding, or other) along with an indicator of whether it was the first or subsequent administration of a vaccine. Producers were also asked to identify the top factors considered when deciding to vaccinate suckling calves and select herd vaccine protocols. A copy of the survey is available from the corresponding author on request.

To facilitate the survey completion producers were encouraged to consult their records, expense receipts and veterinarians, if needed, for details. They were further provided with a handbook listing and describing bovine vaccines, with associated color photographs of product packaging for vaccines currently approved for use in beef cattle in Canada as an aid to recall. The handbook was developed in consultation with practicing beef cattle veterinarians.

Herd attributes including calving, health and productivity data for the 2020 breeding to weaning season were collected in June and December of 2020 using a survey adapted from a productivity survey previously tested and proven with Canadian cow-calf producers (35). Data from this survey were extracted and integrated with the results from the vaccine use survey.

### 2.2. Participant recruitment and survey distribution

All cow-calf producers participating in the Canadian Cow-Calf Surveillance Network (C3SN), a national cattle health and productivity surveillance network established in 2018, were sent the survey. Eligibility requirements for C3SN included: a breeding herd size of ≥40 animals, maintenance of calving records, routine pregnancy testing, and access to email. Herds were recruited through veterinarians, provincial producer groups and social media (36). In July 2021, vaccination surveys were mailed to 162 participating herds across Canada.

### 2.3. Data management and statistical analysis

Responses were entered into a commercial spreadsheet program and checked for accuracy by having a second person review data entry and through logical checks. Vaccine trade names as reported by producers were linked to a list of vaccines licensed for use in Canadian cattle according to the Compendium of Veterinary Products (37) to determine each of the vaccine’s target components. Additional product details were obtained, if necessary, directly from product packaging and labels. Data from the vaccine use survey were merged with producer attribute data and production records (Microsoft Access; Microsoft, St. Louis, Missouri, United States).

Factors potentially associated with vaccine use, including cow herd size (small: <100 animals, medium: 100–300 animals, large: >300 animals), producer age (<40 years), and producer education level (post-secondary vs. high school), were investigated in a series of unconditional (univariate) analysis using logistic regression (StataCorp LP, College Station, Texas).

Analysis of associations of interest between vaccine use and productivity data were investigated using generalized estimating equations to account for clustering of outcomes within herd, a logit link function, binomial distribution, and exchangeable covariance structure (SAS for Windows ver 9.4, SAS Institute, Cary NC). Counts of the outcomes of interest for each herd were included as the numerator and the total numbers of animals at risk in each herd as the denominator adjusting for cow herd size, calving month and geographical location as potential confounders based on a recent data from this region (35). Associations were reported as odds ratios (OR) with 95% confidence intervals; *p* < 0.05 was considered statistically significant.

## 3. Results

### 3.1. Study population

Herds enrolled from British Columbia (6), Alberta (38), Saskatchewan (27), and Manitoba (18) were identified as being from western Canada (69% (91/131)), whereas herds from Ontario (20), Quebec (16), Nova Scotia (2) and New Brunswick (2) were identified as being from eastern Canada (31% (40/131)). Herds were described as primarily commercial (60%, 79/131), primarily purebred/ seedstock (4%, 5/131), and mixed commercial/purebred (36%, 47/131).

The mean number of breeding cows per herd was 193 (median 130, 5th and 95th percentile 40, 527). The mean number of breeding cows per herd in western operations was 244 (median 190, 5th and 95th percentile 64, 644) and 76 (median 65, 5th and 95th percentile 33, 162) in eastern herds. On average, operations had 34 (median 20, 5th and 95th percentile 1, 105) bred heifers and producers purchased in 7 (median 0, 5th and 95th percentile 0, 30) replacement breeding females during the 2020 calendar year.

Twenty-two percent of herds (28/130) were managed by at least one person under the age of thirty and 47% (61/130) under forty. Thirty percent (38/128) of herd managers reported a university degree, 21% (27/128) a college diploma, 18% (23/128) a professional trade, 15% (19/128) a graduate degree, and 16% (21/128) a high school diploma. Denominators differed for producer characteristics as some producers declined to answer specific questions.

Many operations were diversified with other beef production operations; 55% (72/131) reported backgrounding calves, 25% (33/131) maintained stockers or grassers, and 15% (13/131) operated a feedlot. Thirty-four percent (44/131) of operations were strictly cow-calf. More than half of the 2020 calf crop was retained for at least 2 months post weaning in 53% (69/131) of herds. Forty-five (31/69) percent of herds that retained calves had some seedstock. Calving start dates varied and included January (24%, 31/131), February (14%, 18/131), March (25%, 33/131) and April (24%, 31/131). Reported primary calving facilities included pastures (53%, 69/131), corrals or dry lots (40%, 53/131), and barns or covered sheds (27%, 36/131). Community pastures were used by 21% (27/131) herds.

### 3.2. Vaccine use in bulls, cows, and replacement heifers

Most producers vaccinated bulls (83%), cows (97%) and replacement heifers (95%) with at least one vaccine between January 1 and December 31, 2020 (Table 1). Cows and replacement heifers from 92% of herds were vaccinated for BVDV, IBR, BRSV and PI3, with at least half of herds also vaccinating both groups for viral calf scours (coronavirus, rotavirus) (Table 1). However, 8% of producers did not administer BVDV/IBR/BRSV/PI3 vaccines to breeding females and only 17% of producers provided a second scours vaccine to heifers (Table 1). The percent of herds that vaccinated bulls for BVDV, IBR, BRSV and PI3 was lower than for cows. Vaccines for other reproductive diseases were reported less frequently for breeding stock, with replacement heifers most vaccinated against *Leptospira* spp. (36%) and *Campylobacter fetus* (23%).

**Table 1:** Summary of vaccines and booster doses administered to bulls, cows and replacement heifers from January 1 to December 31, 2020 in Canadian cow-calf herds reported as proportion of herds (and number of herds) (*n* = 131 herds).

Vaccine target	Bulls		Cows		Replacement heifers	
	First vaccine	Second vaccine	First vaccine	Second vaccine	First vaccine	Second vaccine
BVDV Type 1 and 2, IBR, BRSV and PI3	0.75 (98)	0.03 (4)	0.92 (121)	0.05 (6)	0.92 (120)	0.18 (23)
*Mannheimia haemolytica*	0.08 (11)	0 (0)	0.04 (5)	0 (0)	0.08 (10)	0 (0)
*Pasteurella multocida*	0.02 (2)	0 (0)	0 (0)	0 (0)	0.01 (1)	0 (0)
*Histophilus somni*	0.16 (21)	0 (0)	0.18 (24)	0.02 (2)	0.22 (29)	0.05 (7)
*Campylobacter fetus*	0.18 (23)	0 (0)	0.21 (28)	0.02 (2)	0.23 (30)	0.05 (7)
*Leptospira* spp.	0.27 (35)	0.01 (1)	0.35 (46)	0.04 (5)	0.36 (47)	0.08 (11)
Coronavirus	0 (0)	0 (0)	0.50 (66)	0.07 (9)	0.53 (70)	0.24 (31)
Rotavirus	0 (0)	0 (0)	0.50 (66)	0.07 (9)	0.53 (70)	0.24 (31)
*Escherichia coli*	0 (0)	0 (0)	0.44 (58)	0.07 (9)	0.47 (62)	0.17 (22)
*Cl. perfringens* (scours)	0 (0)	0 (0)	0.05 (7)	0.02 (2)	0.06 (8)	0.04 (5)
Clostridial vaccine (any)	0.50 (65)	0.02 (3)	0.59 (77)	0.02 (2)	0.66 (86)	0.09 (12)
*Cl.* 7-way^a^	0.08 (11)	0 (0)	0.07 (9)	0 (0)	0.09 (12)	0.01 (1)
*Cl.* 8-way^b^	0.15 (19)	0 (0)	0.16 (21)	0.01 (1)	0.21 (28)	0.02 (3)
*Cl.* 8-way (*Cl. tetani*)^c^	0.17 (22)	0.02 (3)	0.19 (25)	0 (0)	0.23 (30)	0.03 (5)
*Cl.* 9-way^d^	0.12 (16)	0 (0)	0.14 (18)	0.01 (1)	0.17 (22)	0.02 (3)
*Cl. Tetani*	0.29 (38)	0.02 (3)	0.33 (43)	0.01 (1)	0.40 (52)	0.06 (8)
Anthrax	0.02 (3)	0 (0)	0.02 (3)	0 (0)	0.02 (3)	0 (0)
*Fusobacterium*	0.31 (41)	0.02 (3)	0.01 (1)	0 (0)	0 (0)	0 (0)
*Moraxella bovis*	0.02 (2)	0 (0)	0.02 (2)	0 (0)	0.01 (1)	0 (0)
Papillomavirus	0 (0)	0 (0)	0 (0)	0 (0)	0 (0)	0 (0)
Proportion (n) of herds reporting use of any vaccin**e**	**0.83** (109)	**0.05** (7)	**0.97** (127)	**0.10** (13)	**0.95** (124)	**0.34** (44)

Bold values mean overall vaccination levels of that animal class (not significant).

a 7-way contains *Cl. chauvoei*, *Cl. novyi*, *Cl. perfringens* Types B, C, D, *Cl. septicum*, *Cl. sordellii*.

b 8-way contains 7-way plus *Cl. haemolyticum*.

c 8-way (*Cl. tetani*) contains 7-way plus *Cl. haemolyticum* (*Cl. tetani* replaces *Cl. sordellii*).

d 9-way contains 7-way plus *Cl. haemolyticum* and *Cl. Tetani*.

Two-thirds of producers vaccinated their replacement heifers against clostridial disease, while only half vaccinated their cows and bulls during the year (Table 1). Eight-way products were the most common clostridial vaccines reported for bulls, cows and replacement heifers (Table 1). Footrot (*Fusobacterium*) vaccine was administered to bulls in 31% of herds; while only one herd vaccinated cows (Table 1).

Vaccination timing in bulls, cows and replacement heifers was dependent on vaccine type (Table 2). More than half of producers vaccinated cows and most vaccinated replacement heifers against viral pathogens prior to breeding using a MLV product (Table 2). Modified live viral vaccines were most often administered prior to breeding, and inactivated vaccines were slightly more commonly administered at pregnancy testing. Twenty of the 22 herds (91%) that administered a MLV IBR/BVDV vaccine to replacement heifers at pregnancy checking had previously administered two doses of a MLV IBR/BVDV vaccine, and the two other herds administered one prior MLV IBR/BVDV vaccine. Apart from anthrax vaccines (nonencapsulated live culture), all other bacterial vaccines administered to cows and replacement heifers were inactivated. *Campylobacter* and *Leptospira* vaccines were commonly administered prior to breeding (Table 2). Producers who sent cows to community pasture were more likely to administer a *Campylobacter* vaccine (41%, 11/27), than those who did not use community pasture (16%, 17/104) (*p* < 0.01). Cows were vaccinated for *Clostridia* spp. prior to breeding (21%), at pregnancy checking (22%), or before calving (15%). Heifers were vaccinated for clostridial disease prior to breeding in a higher proportion of herds than cows (Table 2).

**Table 2:** Timing of vaccination and vaccine type administered to bulls, cows, and replacement heifers for common vaccine targets used in 131 Canadian cow-calf herds.

Vaccine timing	BVDV	IBR, BRSV, and PI3	*Mh* +/− *Pm*^a^	*H. somni*	*Clostridia* spp.	Calf scours^b^	*Campylobacter* or *Leptospira* spp.	Footrot
Bulls								
Before breeding	0.60 (78)	0.60 (78)	0.04 (5)	0.15 (19)	0.40 (53)	0 (0)	0.24 (32)	0.31 (41)
After breeding	0.15 (20)	0.15 (20)	0.03 (4)	0.02 (2)	0.07 (9)	0 (0)	0.05 (7)	0 (0)
Modified live vaccine	0.52 (68)	0.67 (88)	n/a	n/a	n/a	n/a	n/a	n/a
Before breeding	0.52 (68)	0.54 (71)	n/a	n/a	n/a	n/a	n/a	n/a
After breeding	0.11 (15)	0.13 (17)	n/a	n/a	n/a	n/a	n/a	n/a
Inactivated/killed vaccine	0.11 (15)	0.08 (10)	0.07 (9)	0.16 (21)	0.51 (67)	n/a	0.30 (39)	0.31 (41)
Before breeding	0.08 (10)	0.05 (7)	0.04 (5)	0.15 (19)	0.40 (53)	0 (0)	0.24 (32)	0.31 (41)
After breeding	0.04 (5)	0.02 (3)	0.03 (4)	0.02 (2)	0.07 (9)	0 (0)	0.05 (7)	0 (0)
Cows								
Before breeding	0.54 (71)	0.54 (71)	0.02 (2)	0.11 (14)	0.21 (28)	0.01 (1)	0.25 (33)	0.01 (1)
At pregnancy testing	0.27 (36)	0.28 (37)	0.01 (1)	0.03 (4)	0.22 (29)	0.12 (16)	0.08 (11)	0 (0)
Before calving	0.14 (18)	0.21 (28)	0.01 (1)	0.05 (7)	0.15 (20)	0.44 (57)	0.08 (10)	0 (0)
Modified live vaccine	0.76 (99)	0.84 (110)	n/a	n/a	n/a	n/a	n/a	n/a
Before breeding	0.51 (67)	0.53 (70)	n/a	n/a	n/a	n/a	n/a	n/a
At pregnancy testing	0.13 (16)	0.19 (24)	n/a	n/a	n/a	n/a	n/a	n/a
Before calving	0.10 (13)	0.19 (25)	n/a	n/a	n/a	n/a	n/a	n/a
Inactivated/killed vaccine	0.21 (28)	0.13 (17)	0.04 (5)	0.18 (24)	0.59 (77)	0.53 (69)	0.39 (51)	0.01 (1)
Before breeding	0.03 (4)	0.01 (1)	0.02 (2)	0.11 (14)	0.21 (28)	0.01 (1)	0.25 (33)	0.01 (1)
At pregnancy testing	0.15 (20)	0.10 (13)	0.01 (1)	0.03 (4)	0.22 (29)	0.12 (16)	0.08 (11)	0 (0)
Before calving	0.04 (5)	0.02 (3)	0.01 (1)	0.05 (7)	0.15 (20)	0.44 (57)	0.08 (10)	0 (0)
Replacement heifers								
Before breeding	0.82 (108)	0.81 (106)	0.05 (7)	0.19 (25)	0.46 (60)	0.02 (2)	0.34 (45)	0 (0)
At pregnancy testing	0.21 (28)	0.20 (26)	0.02 (3)	0.04 (5)	0.22 (29)	0.28 (37)	0.06 (8)	0 (0)
Before calving	0.07 (9)	0.11 (14)	0 (0)	0.02 (3)	0.14 (18)	0.50 (66)	0.02 (3)	0 (0)
Modified live vaccine	0.85 (111)	0.89 (117)	0.01 (1)	n/a	n/a	n/a	n/a	n/a
Before breeding	0.76 (100)	0.79 (103)	0.01 (1)	n/a	n/a	n/a	n/a	n/a
At pregnancy testing	0.13 (17)	0.17 (22)	0 (0)	n/a	n/a	n/a	n/a	n/a
Before calving	0.05 (6)	0.09 (12)	0 (0)	n/a	n/a	n/a	n/a	n/a
Inactivated/killed vaccine	0.16 (20)	0.10 (13)	0.07 (9)	0.22 (29)	0.66 (86)	0.55 (72)	0.40 (52)	0 (0)
Before breeding	0.06 (8)	0.04 (5)	0.05 (6)	0.19 (25)	0.46 (60)	0.02 (2)	0.34 (45)	0 (0)
At pregnancy testing	0.08 (11)	0.05 (7)	0.02 (3)	0.04 (5)	0.22 (29)	0.28 (37)	0.06 (8)	0 (0)
Before calving	0.02 (3)	0.02 (2)	0 (0)	0.02 (3)	0.14 (18)	0.50 (66)	0.02 (3)	0 (0)

Reported as proportion of herds that vaccinated (number of herds).

a *Mannheimia haemolytica* with or without *Pasteurella multocida*.

b Scours: BCoV, BRV, *E. coli*, and/or *Clostridium perfringens*.

c *Campylobacter fetus* or *Leptospira* spp.; n/a = not applicable: no registered MLV vaccine available for listed bacterial target.

Bulls from most herds were vaccinated before breeding (Table 2). Modified live BVDV, IBR, BRSV, PI3 vaccines were used in bulls more commonly than inactivated products. Two producers administered a commercially available parenteral (SQ) vaccine containing avirulent live cultured *P. multocida* and *M. haemolytica* to their bulls.

### 3.3. Vaccine use in calves

All calves, nursing and weaned considered together, received at least one vaccination in 99% of herds and were administered at least a second dose of one type of vaccine in 82% of herds (Table 3). The most common vaccine targets were IBR, BRSV, PI3, BVDV, *Clostridia* spp. and *M. haemolytica* (Table 3). The vaccines most likely to be followed by a second administration of a vaccine to the same target in calves, either before, at or after weaning were IBR, BRSV, and PI3 (77%), BVDV, (61%), clostridial vaccines (46%), and *M. haemolytica* (28%) (Table 3). Modified-live vaccines were used almost exclusively in calves for viral respiratory targets. Calves were administered at least one IN IBR/BRSV/PI3 vaccine prior to weaning in 33% (43/131) of herds. Bacterial respiratory vaccines including *M. haemolytica*, *H. somni* and *P. multocida* were administered to calves in 62, 43 and 19% of herds, respectively. Thirty-four percent of all calves received at least one dose of a clostridial vaccine containing tetanus (Table 3).

**Table 3:** Summary of initial vaccine dose and type and booster doses administered to suckling and weaned calves from January 1 to December 31, 2020 in Canadian cow-calf herds reported as proportion of herds (and number of herds).

Vaccine target	All calves (*n* = 131)		Suckling calves (*n* = 131)		Weaned calves					
					All herds (*n* = 131)		Retained^a^ (*n* = 69)		Did not retain (*n* = 62)	
	First vaccine	Second vaccine	First vaccine	Second vaccine	First vaccine	Second vaccine	First vaccine	Second vaccine	First vaccine	Second vaccine
BVDV Type 1 & 2	0.93 (122)	0.61 (80)	0.78 (102)	0.30 (39)	0.57 (75)	0.05 (6)	0.65 (45)	0.07 (5)	0.48 (30)	0.02 (1)
IBR, BRSV & PI3	0.95 (125)	0.77 (101)	0.92 (120)	0.47 (61)	0.58 (76)	0.05 (6)	0.67 (46)	0.07 (5)	0.48 (30)	0.02 (1)
*M. haemolytica*	0.62 (81)	0.28 (37)	0.56 (73)	0.27 (36)	0.30 (39)	0.02 (3)	0.39 (27)	0.04 (3)	0.19 (12)	0 (0)
*P. multocida*	0.19 (25)	0.16 (21)	0.18 (23)	0.04 (5)	0.05 (6)	0.01 (1)	0.04 (3)	0.01 (1)	0.05 (3)	0 (0)
*H. somni*	0.43 (56)	0.16 (21)	0.40 (52)	0.08 (10)	0.16 (21)	0.08 (11)	0.22 (15)	0.13 (9)	0.08 (5)	0.03 (2)
*Campylobacter fetus*	0.02 (3)	0 (0)	0.02 (2)	0 (0)	0.02 (2)	0 (0)	0.01 (1)	0 (0)	0.02 (1)	0 (0)
*Leptospira* spp.	0.07 (9)	0.02 (3)	0.03 (4)	0.01 (1)	0.06 (8)	0.02 (2)	0.07 (5)	0.01 (1)	0.05 (3)	0.02 (1)
Coronavirus	0.06 (8)	0.01 (1)	0.06 (8)	0.01 (1)	0 (0)	0 (0)	0 (0)	0 (0)	0 (0)	0 (0)
Rotavirus	0.06 (8)	0.01 (1)	0.06 (8)	0.01 (1)	0 (0)	0 (0)	0 (0)	0 (0)	0 (0)	0 (0)
*Escherichia coli*	0.02 (2)	0.01 (1)	0.02 (2)	0.01 (1)	0 (0)	0 (0)	0 (0)	0 (0)	0 (0)	0 (0)
*Cl. perfringens*	0.02 (2)	0.01 (1)	0.02 (2)	0.01 (1)	0 (0)	0 (0)	0 (0)	0 (0)	0 (0)	0 (0)
Clostridial vaccine	0.88 (115)	0.46 (60)	0.87 (114)	0.26 (34)	0.27 (36)	0.02 (2)	0.33 (23)	0.03 (2)	0.21 (13)	0 (0)
*Cl.* 7-way^b^	0.27 (36)	0.21 (27)	0.27 (36)	0.08 (10)	0.20 (26)	0 (0)	0.23 (16)	0 (0)	0.16 (10)	0 (0)
*Cl.* 8-way^c^	0.27 (35)	0.11 (15)	0.27 (35)	0.10 (13)	0.02 (2)	0 (0)	0.03 (2)	0 (0)	0 (0)	0 (0)
*Cl.* 8-way (*Cl. tetani*)^d^	0.21 (28)	0.05 (6)	0.21 (28)	0.05 (6)	0 (0)	0 (0)	0 (0)	0 (0)	0 (0)	0 (0)
*Cl.* 9-way^e^	0.13 (17)	0.09 (12)	0.12 (16)	0.04 (5)	0.06 (8)	0.02 (2)	0.07 (5)	0.03 (2)	0.05 (3)	0 (0)
*Cl. tetani*	0.34 (45)	0.14 (18)	0.34 (44)	0.08 (11)	0.06 (8)	0.02 (2)	0.07 (5)	0.03 (2)	0.05 (3)	0 (0)
Anthrax	0.02 (2)	0 (0)	0.02 (2)	0 (0)	0 (0)	0 (0)	0 (0)	0 (0)	0 (0)	0 (0)
*Fusobacterium*	0 (0)	0 (0)	0 (0)	0 (0)	0 (0)	0 (0)	0 (0)	0.01 (1)	0 (0)	0 (0)
*Moraxella bovis*	0.02 (3)	0 (0)	0.02 (2)	0 (0)	0.01 (1)	0 (0)	0.01 (1)	0 (0)	0 (0)	0 (0)
Papillomavirus	0 (0)	0 (0)	0 (0)	0 (0)	0 (0)	0 (0)	0 (0)	0 (0)	0 (0)	0 (0)
Proportion (n) of herds reporting use of any vaccine	**0.99** (130)	**0.82** (108)	**0.98** (129)	**0.56** (74)	**0.66** (87)	**0.11** (15)	**0.77** (53)	**0.17** (12)	**0.55** (34)	**0.05** (3)

Bold values mean overall vaccination levels of that animal class (not significant).

a Producer retained >50% of the calf herd for ≥2 months post weaning.

b 7-way contains *Cl. chauvoei*, *Cl. novyi*, *Cl. perfringens* Types B, C, D, *Cl. septicum, Cl. sordellii*.

c 8-way contains 7-way plus *Cl. haemolyticum*.

d 8-way (*Cl. tetani*) contains 7-way plus *Cl. haemolyticum* (*Cl. tetani* replaces *Cl. sordellii*).

e 9-way contains 7-way plus *Cl. haemolyticum* and *Cl. Tetani*.

Suckling calves received at least one vaccine in 98% of herds and the most common targets were IBR, BRSV, and PI3 (92%) as well as *Clostridia* spp. (87%), BVDV (78%), and *M. haemolytica* (56%) (Table 3). Most suckling calves were vaccinated after two weeks of age; however, the most common vaccine antigens administered prior to 2 weeks of age were for IBR/BRSV/PI3 (21%, 27/131) (Table 4).

**Table 4:** Vaccination timing and vaccine type for all vaccine doses administered to weaned and suckling calves for common vaccine targets used in 131 Canadian cow-calf herds.

Vaccination timing	BVDV types 1 or 2	IBR, BRSV, and PI3	*Mh* +/− *Pm* ^a^	*H. somni*	*Clostridia* spp.
Suckling calves					
Birth – 2 weeks	0 (0)	0.21 (27)	0.07 (9)	0 (0)	0.04 (5)
2 weeks – 3 months	0.50 (65)	0.60 (79)	0.42 (55)	0.31 (40)	0.67 (88)
>3 months	0.52 (68)	0.62 (81)	0.40 (53)	0.23 (30)	0.42 (55)
Modified live vaccine	0s.75 (98)	0.91 (119)	0.18 (23)	n/a	n/a
Birth – 2 weeks	(0)	0.20 (26)	0.07 (9)	n/a	n/a
2 weeks – 3 months	0.49 (64)	0.60 (78)	0.08 (11)	n/a	n/a
>3 months	0.50 (65)	0.62 (81)	0.08 (10)	n/a	n/a
Inactivated/killed vaccine	0.03 (4)	0.02 (2)	0.47 (61)	0.40 (52)	0.87 (114)
Birth – 2 weeks	0 (0)	0.01 (1)	0.01 (1)	0 (0)	0.04 (5)
2 weeks – 3 months	0.01 (1)	0.01 (1)	0.34 (44)	0.31 (40)	0.67 (88)
>3 months	0.02 (3)	0 (0)	0.33 (43)	0.23 (30)	0.42 (55)
Intranasal vaccine	n/a	0.33 (43)	0.10 (13)	n/a	n/a
Birth – 2 weeks	n/a	0.20 (26)	0.07 (9)	n/a	n/a
2 weeks – 3 months	n/a	0.14 (18)	0.03 (4)	n/a	n/a
>3 months	n/a	0.03 (4)	0.01 (1)	n/a	n/a
Weaned calves					
At weaning	0.40 (52)	0.40 (52)	0.20 (26)	0.16 (21)	0.20 (26)
After weaning	0.27 (35)	0.23 (30)	0.16 (21)	0.08 (11)	0.09 (12)
Modified live vaccine	0.56 (73)	0.57 (75)	0.05 (6)	n/a	n/a
At weaning	0.38 (50)	0.41 (54)	0.02 (3)	n/a	n/a
After weaning	0.26 (34)	0.21 (28)	0.02 (3)	n/a	n/a
Inactivated/killed vaccine	0.02 (3)	0.02 (2)	0.30 (39)	0.27 (21)	0.27 (36)
At weaning	0.02 (2)	0.01 (1)	0.18 (23)	0.16 (21)	0.20 (26)
After weaning	0.01 (1)	0.02 (2)	0.14 (18)	0.08 (11)	0.09 (12)
Intranasal vaccine	n/a	0.02 (3)	0 (0)	n/a	n/a
At weaning	n/a	0.02 (3)	0 (0)	n/a	n/a
After weaning	n/a	0.01 (1)	0 (0)	n/a	n/a

Reported as proportion of herds that vaccinated (number of herds).

a *Mannheimia haemolytica* with or without *Pasteurella multocida*; n/a = not applicable: no registered MLV vaccine available for listed bacterial target.

Sixty-six percent of producers (87/131) vaccinated calves at or after weaning, most commonly against IBR/BRSV/PI3, BVDV and *M. haemolytica* (Table 3). Producers who retained >50% of their calf crop for ≥2 months post weaning administered IBR/BRSV/PI3 vaccines to calves at or after weaning (67%, 46/69) more often than those who did not retain calves (48%, 30/62) (*p* < 0.05).

**Figure 1:**
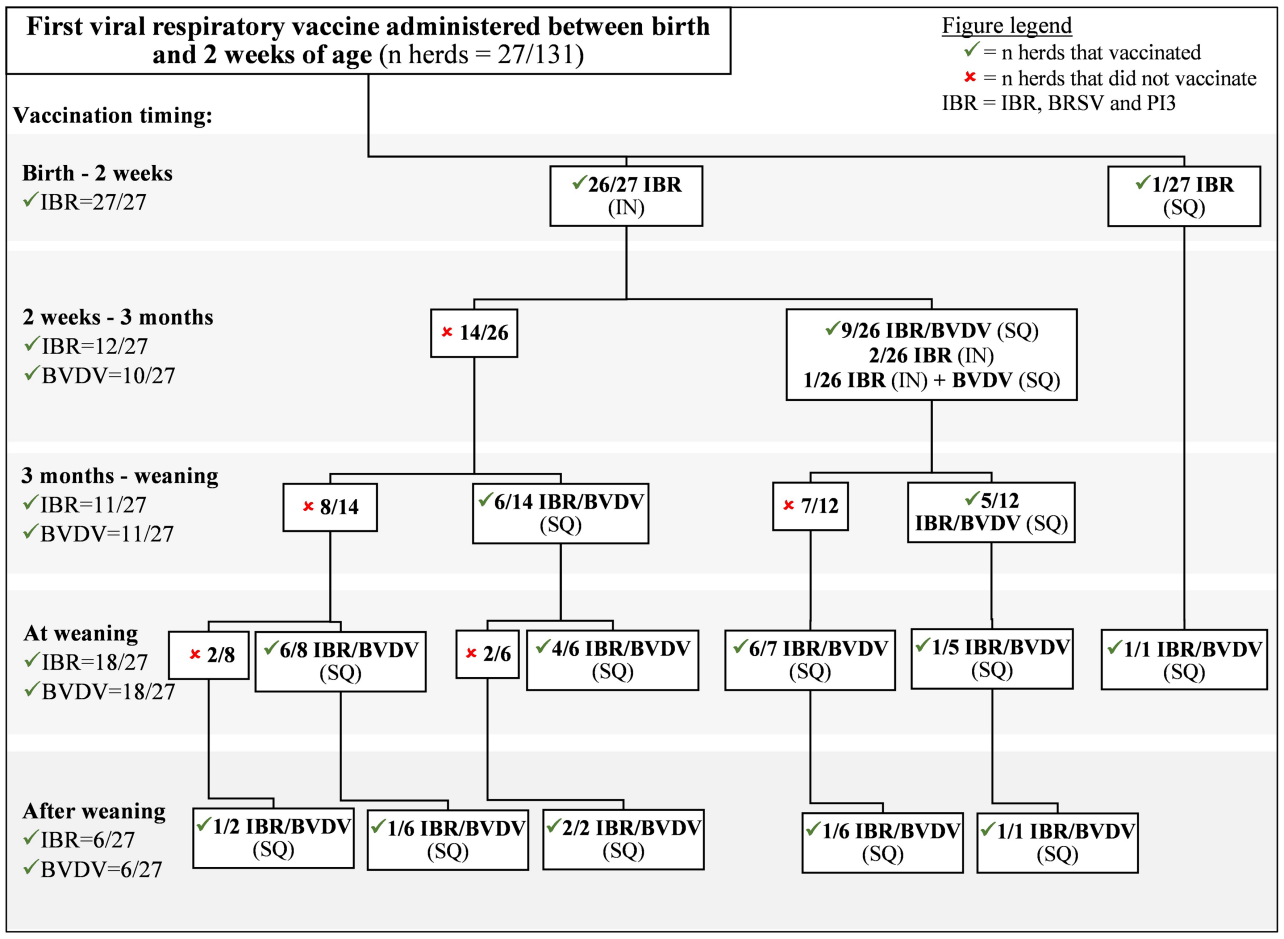
Viral respiratory vaccine protocols of Canadian cow-calf producers who first vaccinated calves against IBR, BVDV and PI3 between birth and 2 weeks of age.

**Figure 2:**
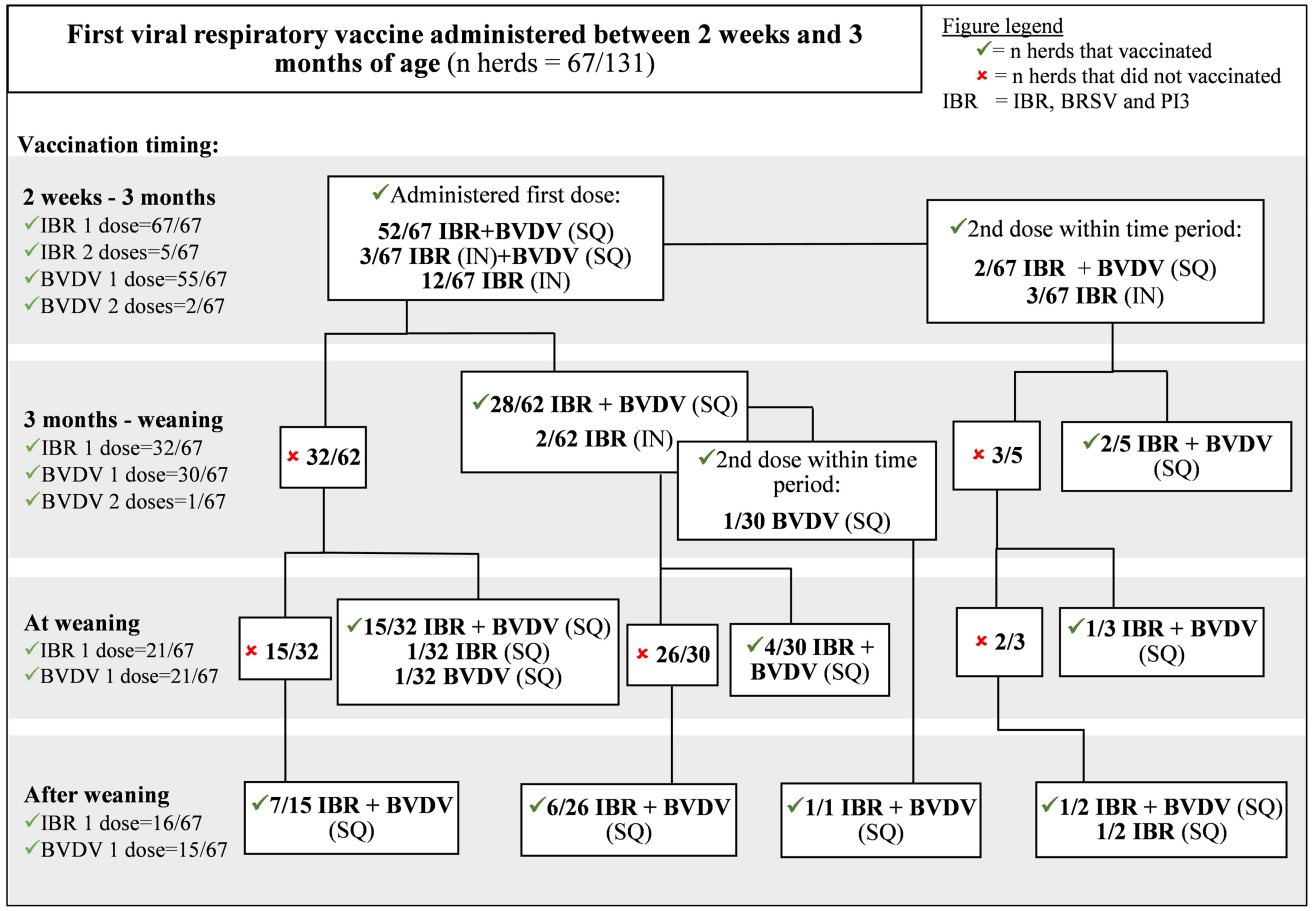
Viral respiratory vaccine protocols of Canadian cow-calf producers who first vaccinated calves against IBR, BVDV and PI3 between 2 weeks and 3 months of age.

**Figure 3:**
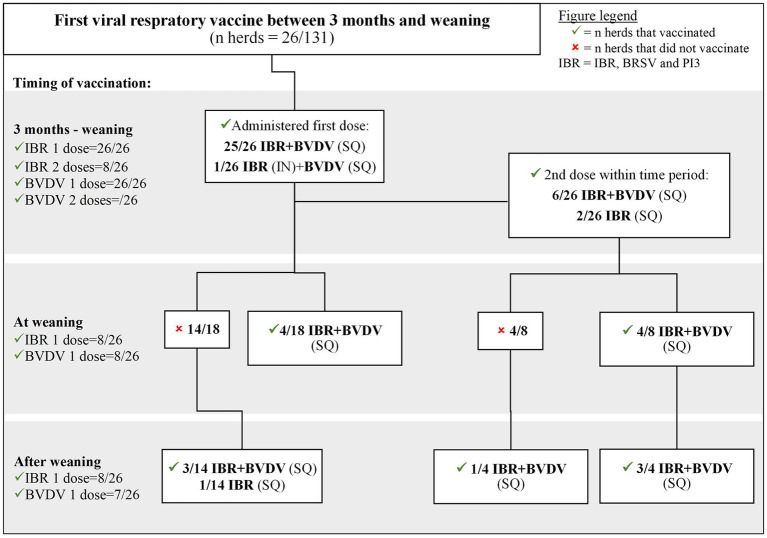
Viral respiratory vaccine protocols of Canadian cow-calf producers who first vaccinated calves against IBR, BVDV and PI3 between 3 months of age and weaning.

Vaccination protocols for IBR/BRSV/PI3 and BVDV were assembled into flow charts based on timing of first dose administered (Figures 1–3). Twenty-one (27/131) percent of producers administered IBR/BRSV/PI3 vaccines to calves before 2 weeks of age (26 intranasal [IN], 1 subcutaneous [SQ]) (Figure 1). BVDV vaccines were not administered prior to 2 weeks of age. Sixty-seven percent (18/27) of herds that vaccinated against IBR/BRSV/PI3 before 2 weeks of age also received a second IBR/BRSV/PI3 dose prior to weaning. Of the 27 herds vaccinated against IBR/BRSV/PI3 prior to 2 weeks of age, 59% (16/27) vaccinated against BVDV and 19% (5/27) provided a second BVDV dose prior to weaning.

Fifty-one percent (67/131) of producers administered initial viral respiratory vaccines between 2 weeks and 3 months of age; 12 IN IBR/BRSV/PI3, 3 IN IBR/BRSV/PI3 with SQ BVDV, and 52 SQ IBR/BRSV/PI3/BVDV (Figure 2). Fifty-two percent (35/67) of herds that were administered IBR/BRSV/PI3 vaccines between 2 weeks and 3 months were also administered a second IBR/BRSV/PI3 vaccine prior to weaning. Most herds administered a BVDV vaccine (88%, 59/67) and 45% (30/67) administered a second BVDV vaccine dose prior to weaning.

There was no significant difference in whether a herd would receive a second dose of IBR/BRSV/PI3 vaccine (*p* = 0.25) before weaning if the calves were vaccinated for IBR/BRSV/PI3 for the first time between birth and 2 weeks or 2 weeks and 3 months. However, herds where calves were vaccinated with IBR/BRSV/PI3 vaccine before 2 weeks of age were less likely (*p* = 0.04) than those vaccinated from 2 weeks to 3 months to administer a second BVDV vaccine dose.

Twenty percent (26/131) of calves were administered initial SQ IBR/BRSV/PI3 and BVDV vaccines between 3 months and weaning (Figure 3). Of those, 23% (6/26) were administered a second SQ IBR/BRSV/PI3 and BVDV vaccine, and 8% (2/26) were administered a second SQ IBR/BRSV/PI3 vaccine prior to weaning. Calves were more likely to receive a second dose of IBR/BRSV/PI3 vaccine (*p* = 0.03), but not BVDV vaccine (*p* = 0.47), before weaning if the calves were vaccinated for IBR/BRSV/PI3 for the first time between birth and 3 months than after 3 months.

Bacterial vaccines including *Clostridia* spp., *M. haemolytica, P. multocida,* and *H. somni* were typically administered to suckling calves after 2 weeks of age (Table 4). Few producers administered a second *M. haemolytica* (27%) or *Clostridia* spp (26%) vaccine dose prior to weaning (Table 3).

Seventy percent (92/131) of producers vaccinated calves against clostridial disease prior to 3 months of age, but only one herd was administered a booster in that period. Twenty-five percent (33/131) administered a second clostridial vaccine between 3 months and weaning. Eighty-seven percent (114/131) of producers vaccinated calves against *Clostridia* spp. between birth and weaning, but only 26% (34/131) of producers administered a second clostridial vaccine before weaning. Eight-way vaccines with (21%, 28/131) and without (27%, 35/131) tetanus were the most common choices in suckling calves. Suckling calves were administered at least one vaccine containing *Cl. tetani* in 34% of herds (Table 3). Seven-way clostridial vaccines were the most common choice for weaned calves (Table 3).

Forty-seven percent of producers (61/131) administered vaccines containing *M. haemolytica* (15%, 20/131 with *P. multocida*), prior to 3 months of age, and four producers also administered a second *M. haemolytica* dose prior to 3 months. Thirty-five percent (46/131) vaccinated against *M. haemolytica* (7%, 9/131 with *P. multocida*) between 3 months of age and weaning. Of those 46 producers, one administered a second dose to calves again prior to weaning, 7 boosted at weaning, and 8 boosted after weaning. Intranasal *M. haemolytica* and *P. multocida* vaccines were administered in 10% (13/131) of herds, and one herd provided 2 doses of an IN *M. haemolytica* and *P. multocida* vaccine prior to weaning. All 9 herds that vaccinated against *M. haemolytica* and *P. multocida* between birth and 2 weeks used an intranasal vaccine.

### 3.4. Factors associated with vaccine use

Significant unconditional odds ratios were reported from logistic regression where cow herd size, retaining >50% of calves for ≥ 2 months after weaning and reporting only commercial vs. some purebred cattle were associated with vaccine use (Table 5). Large herds were more likely to vaccinate cows and heifers against clostridial disease, weaned calves against *M. haemolytica* and clostridial disease, and suckling calves against *H. somni* compared to small herds. Medium sized herds were also more likely to vaccinate heifers and suckling calves against clostridial disease, weaned calves against *M. haemolytica*, and suckling calves against *H. somni* than small herds (Table 5). Weaned calves were more likely to be vaccinated against BVDV, IBR, BRSV, PI3, *M. haemolytica* +/− *P. multocida* in herds that retained most of their calves for more than 2 months (Table 5). Herds that were strictly commercial were less likely to vaccinate weaned calves against BVDV and breeding cattle against bovine genital campylobacteriosis and leptospirosis than herds that contained some seedstock (Table 5).

**Table 5:** Summary of unconditional associations between breeding cow herd size, calf retention and operation type on vaccine use in Canadian cow-calf herds (*n* = 131).

Risk factor/Vaccine use	Herd size	Odds Ratio^a^	95% CI		*p*-value
Breeding cow herd size					
Cows vaccinated against clostridial disease	<100	(Ref)			**0.05** ^**b**^
	100–300	1.86	0.86	4.02	0.12
	>300	3.71	1.25	11.0	**0.02**
Heifers vaccinated against clostridial disease	<100	(Ref)			**0.01** ^**b**^
	100–300	2.32	1.04	5.19	**0.04**
	>300	6.42	1.68	24.5	**<0.01**
Weaned calves vaccinated against *M. haemolytica* +/− *P. multocida*	<100	(Ref)			**<0.01** ^**b**^
	100–300	2.86	1.09	7.51	**0.03**
	>300	5.71	1.84	17.8	**<0.01**
Weaned calves vaccinated against clostridial disease	<100	(Ref)			**0.04** ^**b**^
	100–300	1.93	0.75	4.96	0.17
	>300	2.33	1.36	12.5	**0.01**
Suckling calves vaccinated against *H. somni*	<100	(Ref)			**0.04** ^**b**^
	100–300	2.39	1.04	5.47	**0.04**
	>300	3.45	1.22	9.72	**0.02**
Suckling calves vaccinated against clostridial disease	<100	(Ref)			**0.01** ^**b**^
	100–300	6.51	1.72	24.7	**<0.01**
	>300	3.77	0.77	18.5	0.10
Retained > 50% of 2020 calf crop for ≥ 2 months post weaning vs did not retain > 50% of calf crop					
Heifers vaccinated against BVDV, IBR, BRSV, and PI3		8.81	1.05	73.8	0.05
Weaned calves vaccinated against BVDV		2.43	1.20	4.93	**0.01**
Weaned calves vaccinated against IBR, BRSV and PI3		2.13	1.05	4.32	**0.04**
Weaned calves vaccinated against *M. haemolytica*		2.68	1.21	5.93	**0.02**
Weaned calves vaccinated at least once for any target		2.73	1.29	5.78	**0.01**
100% commercial cow-calf herd vs commercial and purebred or purebred herd					
Weaned calves vaccinated against BVDV		0.43	0.21	0.91	**0.03**
Weaned calves vaccinated against *Campylobacter fetus* and/or *Leptospira* spp.		0.08	0.01	0.67	**0.02**

Bolded characters indicate a statistically significant values.

a Unconditional odds ratio.

b *p*-value for Wald test of categorical variable.

Herds that started calving in February and March were less likely to vaccinate weaned calves against BVDV, IBR, BRSV, and PI3 than herds that started calving in January (Table 6). Herds that calved later in the spring (April and May) were less likely to vaccinate cows against calf scours compared to those that calved in January. Finally, herds that calved in March were more likely to administer IBR/BRSV/PI3 vaccines to suckling calves, compared to those that calved in January (Table 6).

**Table 6:** Unconditional associations between calving month and vaccine use in Canadian cow-calf herds (*n* = 131).

Vaccine use	Calving month	Odds Ratio^a^	Lower 95% CI	Upper 95% CI	*p*-value
Cows vaccinated against calf scours	Jan	(Ref)			**0.02** ^b^
	Feb	0.95	0.28	3.28	0.94
	Mar	0.73	0.26	2.05	0.55
	Apr	0.24	0.08	0.69	**<0.01**
	May	0.05	0.01	0.48	**<0.01**
	Nov-Dec	0.60	0.13	2.71	0.50
Weaned calves vaccinated against BVDV	Jan	(Ref)			**<0.01** ^b^
	Feb	0.19	0.05	0.66	**<0.01**
	Mar	0.17	0.06	0.50	**<0.01**
	Apr	0.58	0.19	1.81	0.35
	May	0.44	0.10	2.00	0.29
	Nov-Dec	0.36	0.08	1.74	0.21
Weaned calves vaccinated against IBR, BRSV, and PI3	Jan	(Ref)			**<0.01** ^b^
	Feb	0.23	0.07	0.82	**0.02**
	Mar	0.15	0.05	0.44	**<0.01**
	Apr	0.68	0.22	2.15	0.51
	May	0.44	0.10	2.00	0.29
	Nov-Dec	0.58	0.12	2.95	0.51
Suckling calves vaccinated against IBR (<3 months of age with injectable product)	Jan	(Ref)			**<0.01** ^b^
	Feb	3.30	0.98	11.1	0.05
	Mar	4.83	1.68	13.9	**<0.01**
	Apr	2.75	0.97	7.80	0.06
	May	0.53	0.09	2.94	0.46
	Nov-Dec	0.26	0.03	2.40	0.24

Bolded characters indicate a statistically significant values.

a Unconditional odds ratio.

b *p*-value for Wald test of categorical variable.

### 3.5. Regional vaccination trends

Generally, reported vaccination practices were consistent between eastern and western herds with a few differences (Table 7). Western herds were substantially more likely to administer clostridial vaccines to all classes of cattle. Eastern herds were more likely to vaccinate cows for *M. haemolytica* +/− *P. multocida,* but only 1 western and 4 eastern herds reported use of this vaccine. While 44% of western producers administered footrot vaccines to their bulls, eastern herds did not report use of this vaccine. Western producers were more likely to vaccinate calves either before or after weaning against *M. haemolytica* and *P. multocida*, and suckling calves for *H. somni* than eastern producers. Thirty-one percent of western herds (28/91) and 38% of eastern herds (15/40) administered at least one IN IBR/BRSV/PI3 vaccine to calves prior to weaning.

**Table 7:** Relative difference between Western and Eastern regions of Canada and vaccines used in cow-calf herds.

Administered ≥1 dose	Western herds (*n* = 91)	Eastern herds (*n* = 40)	OR^a^	95% CI	*p*-value
Bulls					
Any vaccine	0.82 (75)	0.85 (34)	0.83	0.30, 2.30	0.72
BVDV Type 1 or 2	0.73 (66)	0.80 (32)	1.24	0.28, 5.50	0.78
IBR, BRSV, PI3	0.73 (66)	0.80 (32)	1.24	0.28, 5.50	0.78
*M. haemolytica +/− P. multocida*	0.09 (8)	0.08 (3)	1.35	0.34, 5.46	0.67
*H. somni*	0.20 (18)	0.08 (3)	3.04	0.84, 11.0	0.09
*Campylobacter* and/or *Leptospira* spp.	0.27 (25)	0.35 (14)	0.82	0.35, 1.88	0.63
*Clostridia* spp.	0.57 (52)	0.33 (13)	2.77	1.27, 6.05	**0.01**
Footrot	0.44 (40)	0 (0)	–	–	**<0.01**
Cows					
Any vaccine	0.96 (87)	1.00 (40)			
BVDV Type 1 or 2	0.92 (84)	0.93 (37)	0.97	0.24, 3.97	0.97
IBR, BRSV, PI3	0.92 (84)	0.93 (37)	0.97	0.24, 3.97	0.97
*M. haemolytica +/− P. multocida*	0.01 (1)	0.10 (4)	0.10	0.01, 0.93	**0.04**
*H. somni*	0.22 (20)	0.10 (4)	2.54	0.81, 7.97	0.11
*Campylobacter* and/or *Leptospira* spp.	0.33 (30)	0.48 (19)	0.54	0.25, 1.16	0.12
*Clostridia* spp.	0.68 (62)	0.33 (13)	4.44	2.00, 9.83	**<0.01**
Calf scours	0.55 (50)	0.48 (19)	1.35	0.64, 2.84	0.43
Footrot	0.02 (2)	0 (0)	–	–	0.99
Replacement heifers					
Any vaccine	0.97 (88)	0.90 (36)	3.26	0.69, 15.3	0.13
BVDV Type 1 or 2	0.95 (86)	0.85 (34)	3.04	0.87, 10.6	0.08
IBR, BRSV, PI3	0.95 (86)	0.85 (34)	3.04	0.87, 10.6	0.08
*M. haemolytica +/− P. multocida*	0.05 (5)	0.13 (5)	0.41	0.11, 1.49	0.18
*H. somni*	0.26 (24)	0.13 (5)	2.51	0.88, 7.14	0.09
*Campylobacter* and/or *Leptospira* spp.	0.36 (33)	0.43 (17)	0.77	0.36, 1.64	0.50
*Clostridia* spp.	0.82 (75)	0.33 (13)	9.74	4.15, 22.9	**<0.01**
Calf scours	0.59 (54)	0.43 (17)	1.97	0.93, 4.20	0.08
Suckling calves					
Any vaccine	0.98 (89)	1.00 (40)			
BVDV Type 1 or 2	0.80 (73)	0.73 (29)	1.53	0.65, 3.65	0.33
IBR, BRSV, PI3	0.91 (83)	0.93 (37)	0.84	0.21, 3.35	0.81
IN IBR, BRSV, PI3	0.31 (28)	0.38 (15)	0.74	0.34, 1.62	0.45
*M. haemolytica +/− P. multocida*	0.67 (61)	0.30 (12)	4.74	2.12, 10.6	**<0.01**
*H. somni*	0.52 (47)	0.13 (5)	7.48	2.69, 20.8	**<0.01**
*Clostridia* spp.	0.95 (86)	0.70 (28)	7.37	2.39, 22.8	**<0.01**
Weaned calves					
Any vaccine	0.64 (58)	0.73 (29)	0.67	0.29, 1.51	0.33
BVDV Type 1 or 2	0.54 (49)	0.65 (26)	0.63	0.29, 1.36	0.24
IBR, BRSV, PI3	0.54 (49)	0.68 (27)	0.56	0.26, 1.22	0.15
*M. haemolytica +/− P. multocida*	0.35 (32)	0.18 (7)	2.56	1.02, 6.42	**0.05**
*H. somni*	0.18 (16)	0.13 (5)	1.49	0.51, 4.40	0.47
*Clostridia* spp.	0.33 (30)	0.15 (6)	2.79	1.05, 7.36	**0.04**

Bolded characters indicate a statistically significant values.

a Unconditional odds ratio.

Reported as proportion of herds (number of herds).

### 3.6. Motivations for vaccine use

The top three factors that producers considered when deciding what vaccines to use on their operations were the importance of disease in the herd, economic benefits of using the vaccine, and potential to minimize treatment rate and antimicrobial use (Table 8). Eighteen producers (14%) independently identified advice from their veterinarian as a top influencing factor in vaccine choice. Producer’s top three reasons for choosing whether to vaccinate suckling calves were convenience, need for adequate labor to handle calves, and history of calf health problems (Table 8).

**Table 8:** Summary of Canadian cow-calf producer’s top three factors influencing their decisions around whole herd vaccination protocols and whether to vaccinate suckling calves (*n* = 131).

Top three influential factors	Proportion of herds (*n*)
Whole herd vaccination protocols	
Importance of the disease in my herd	0.64 (84)
Economic benefits of using that vaccine in my herd	0.53 (69)
Potential to minimize treatment rate and antimicrobial use	0.52 (68)
Time of year the vaccine needs to be given	0.33 (43)
Whether the vaccine is modified live or killed/inactivated	0.31 (40)
Whether I must boost the vaccine (more than 1 dose needed)	0.21 (28)
Other (*Answers included: veterinarian’s advice, sale protocol*)	0.18 (23)
Route of administration	0.13 (17)
Potential reactions or side effects of the vaccine	0.07 (9)
Vaccine cost	0.06 (8)
Whether I must mix the vaccine before use	0 (0)
Vaccination of suckling calves	
Convenience (calves were being handled for some other reason)	0.77 (101)
Need for adequate labor to handle and vaccinate the calves	0.63 (83)
History of calf health problems	0.41 (54)
Need for adequate handling facilities	0.31 (41)
Busy time of year	0.28 (37)
Challenge of separating cow from calf	0.14 (18)
Other (*Answers included: veterinary recommendation, sale protocol, age of calf, location of cow herd from home, calves born on pasture, weather*)	0.14 (18)
Uncertainty about vaccine effectiveness	0.06 (8)
Cost of the vaccine	0.05 (7)

### 3.7. Vaccine use and associations with calf productivity and pregnancy in breeding females

Calf morbidity, treatment, and mortality rates were available for 124 of the 131 herds in this cohort and vaccine use was examined for association with these outcomes (Table 9). Herds that vaccinated suckling calves against clostridial disease had a lower risk of calf mortality, and herds that vaccinated against BVDV and *M. haemolytica* (+/− *P. multocida*), had a higher risk of pneumonia in calves. No associations were found between vaccine use and risk of calf scours or nonpregnancy in breeding females.

**Table 9:** Associations (*p* < 0.20) between vaccination status and calf mortality, calf morbidity, and nonpregnant breeding females (*n* = 124).

Reported using vaccine (yes vs. no)	Odds Ratio^a^	Lower 95% CI	Upper 95% CI	*p*-value
Calf mortality				
Heifers vaccinated against:				
Calf scours	0.80	0.61	1.05	0.10
Suckling calves vaccinated against:				
Clostridial disease	0.65	0.43	0.97	**0.03**
Calf morbidity due to pneumonia				
Suckling calves vaccinated against:				
BVDV types 1 and 2	2.02	1.17	3.47	**0.01**
*M. haemolytica* +/− *P. multocida*	2.31	1.32	4.07	**<0.01**
*H. somni*	1.59	0.94	2.70	0.08
Any target	2.16	0.88	5.29	0.09
Calf morbidity due to calf scours				
Suckling calves vaccinated against:				
Any target	0.29	0.07	1.30	0.11
Non-pregnancy in breeding females				
Cows vaccinated against:				
*Campylobacter fetus* or *Leptospira* spp.	1.22	0.98	1.52	0.07
Heifers vaccinated against:				
BVDV, IBR, BRSV, and PI3	1.33	0.99	1.78	0.06

a Odds ratio adjusted for cow herd size, calving month, and geographical location.

## 4. Discussion

This paper presents the first detailed picture of vaccine use practices in cow-calf herds from across Canada. Differences in vaccine use between eastern and western herds were limited. Identified differences in clostridial vaccination echoed previous reports where herds in Ontario were less likely to use clostridial vaccines than western herds (25, 33). While regional differences in some vaccines might be due to herd size, facilities, and labor availability, the risk of clostridial diseases is ubiquitous across the country.

Vaccination of the cow herd was higher compared to 2015/2016 reports from eastern provinces (18–20%) but was similar to reports of the same year from western provinces (25, 33). Most producers vaccinated their cows against viruses associated with respiratory and reproductive diseases; however, there are still herds in which these vaccines are not utilized. Less than one-third of producers vaccinated against *Campylobacter* and 35% of herds vaccinated against *Leptospira*. Producers that used community pastures were more likely to vaccinate cows against *Campylobacter* than those that did not. Annual *Campylobacter* and *Leptospira* revaccination are not considered core vaccines by the American Association of Bovine Practitioners, but are recommended for herds considered to be at high risk for either of these infections due to biosecurity challenges, comingling on community pastures or geographic location (19, 20). Waldner et al. reported similar vaccination trends for BVDV in cows, but higher levels of bacterial respiratory vaccines in cows (39).

Vaccination of bulls was less common than for cows and replacement heifers and continues to present an opportunity for improvement. Commonly, bulls were vaccinated against BVDV, IBR, BRSV, PI3, clostridial pathogens, footrot (*Fusobacterium*) and *Leptospira* spp. Most producers followed recommended practices, and vaccinated bulls prior to breeding. According to a survey of US and Canadian beef cattle veterinarians, 79% recommended vaccinating bulls at the same time as the cow herd (19). The proportion of western producers that vaccinated bulls for at least one target was at a similar level to a 2016 study (25); however, the proportion that vaccinated against BVDV, IBR, PI3, BRSV, bovine genital campylobacteriosis, and clostridial diseases seemed to be moderately higher. Compared to eastern Canadian herds in 2015/2016, overall vaccination of bulls was higher by 15–24% (33). It was more common for Canadian herds to administer one or more vaccines to bulls (83%) than US cow-calf herds surveyed in 2016 (44%) (40).

While clostridial vaccines are considered a core vaccine for bulls, only 47% of producers vaccinated their bulls during 2020 (20). Some producers are potentially boosting clostridial vaccines every 2 or more years rather than vaccinating annually. Thus, this survey might have overlooked herds where 2020 was an “off” year for clostridial vaccinations. Clostridial vaccine use was only modestly higher in cows. Although most replacement heifer vaccination practices were similar to that reported for cows, the proportion of herds reporting clostridial vaccine use was higher for heifers than for cows. This might reflect more consistent annual vaccination protocols for heifers. While not all herds report annual use of clostridial vaccines, all clostridial vaccines approved for use in Canada recommend annual revaccination in adult beef cattle. Biannual vaccination with these products is an off-label recommendation.

Replacement heifers were more commonly vaccinated than cows and bulls for all other core vaccines, and these were given almost exclusively as MLV vaccines, prior to breeding. No heifers in this cohort of herds were administered a MLV BVDV/IBR/BRSV/PI3 vaccine at pregnancy testing without at least one prior vaccination of the same kind. More than 9 out of 10 producers using MLV at pregnancy testing provided two prior doses to their heifers as recommended by at least one commonly used commercial vaccine (38). This is a good indication that many producers are aware of the potential risks of MLV vaccines and label recommendations for proper use (20). However, while more replacement heifers than cows were vaccinated for scours or calf diarrhea pathogens, less than half received the second dose recommended by product labels.

Vaccination of both suckling and weaned calves against at least one pathogen has increased since previous reports of herds from western Canada, and northern Ontario and Quebec (25, 34). The potential benefits of vaccinating suckling calves against BRD pathogens particularly for herds with a history of BRD likely influenced vaccine choices (30). Many producers identified the “importance of the disease in the herd” as an influential factor when determining vaccine protocols. The most common vaccines administered to calves were the core vaccines recommended by the AABP (BVDV, IBR, BRSV, PI3 and clostridial vaccines) (20).

Modified-live vaccines were used almost exclusively in calves for all viral targets. Most (90–93%) surveyed North American beef cattle veterinarians recommended the use of MLV vaccines in calves prior to and after weaning (19). According to previous reports of western herds, modified-live BVDV vaccines were used exclusively in both suckling calves and weaned calves (25). Vaccine use differentiated by type (MLV or inactivated/killed) has not been reported for eastern provinces.

Compared to western cow-calf herd vaccination benchmarks reported in 1997 and 1998, the current vaccination landscape is unrecognizable (41). The focus of calf vaccination has shifted to respiratory disease. Compared to 1997/98, vaccination of calves in 2020 was at a higher proportion for all reported targets except for *P. multocida*. This includes BVDV (↑ 53%), IBR (↑49%), PI3 (↑54%), BRSV (↑62%), and *H. somni* (↑7%) (41). Compared to western Canadian herds in 2016, the proportion of herds administered one dose to suckling calves was at a similar proportion; IBR, BRSV and PI3 vaccination was higher (7%), while BVDV vaccination was slightly lower (4%), but the proportion of producers who boosted calves prior to weaning was higher for all viral respiratory targets (4–13%) (25). This is likely attributed to the increased use of intranasal respiratory vaccines, which do not contain BVDV. Use of intranasal viral respiratory vaccines at birth (21%) was higher compared to previous reports for western Canada (9%), Ontario (12%) and Atlantic Canada (14%) (25, 32, 33). Compared to parenteral vaccines, intranasal respiratory vaccines administered in young calves has been shown to produce a better immune priming response in the face of maternal antibodies (13, 14, 42, 43). Protection provided by intranasal respiratory vaccines may be unpredictable and short term (44).

Before weaning, 47% of herds boosted IBR/BRSV/PI3 vaccines, but less than 20% boosted clostridial vaccines. Most commercially available IBR/BRSV/PI3 and clostridial vaccines approved for use in Canadian calves require a second dose after initial vaccination. Calves from herds given an initial IBR/BRSV/PI3 vaccines before 3 months of age were more likely to receive a second vaccine prior to weaning.

Producers identified “convenience (calves were being handled for another reason)” as the top factor influencing their decision to vaccinate nursing calves. Lack of labor, time, access to facilities, weather, or the desire to minimize handling stress might have contributed to limited vaccine boosting in nursing calves. Further, due to a lack of preconditioning incentives for Canadian producers, the largest economic return from optimizing protection due to vaccination is not seen by the cow-calf producer, but rather the in feedlot. Vaccination prior to weaning as part of a preconditioning program has shown to reduce the incidence of respiratory disease and increase average daily gain in the feedlot (26, 27).

Bacterial respiratory vaccines including *M. haemolytica* and *P. multocida* were less common than viral respiratory vaccines which is expected as these vaccines are considered “risk based,” and recommended on an individual herd basis (20). Most herds administered this vaccine after 2 weeks of age and before weaning. Surveyed North American bovine veterinarian’s recommendations for best timing of initial *M. haemolytica* vaccination were inconsistent; recommended times were at branding (45%), before weaning (77%), and after weaning (49%) (19).

The apparent association between vaccination of suckling calves against BVDV or *M. haemolytica* and an increased risk of pneumonia in suckling calves is consistent with previous western Canadian herd surveys that reported beef calves vaccinated for BRD near birth were more likely to be treated for BRD (39). As was noted earlier, producers reported using vaccines in response to the importance of disease in their herds. This association is likely explained by this factor, whereby herds with a historical or ongoing respiratory disease problem were more likely to use these vaccines. Further, in 20% of herds from the current study these vaccines were first administered after 3 months of age after and following the first peak risk for respiratory disease (39).

Conversely, while the reduced risk of calf mortality in suckling calves that were vaccinated against clostridial disease could reflect vaccine success, vaccine use could also potentially reflect overall herd management. One earlier study from western Canada reported the potential benefits of vaccinating cows for clostridial diseases and subsequent protection of the calf crop, and while clostridial vaccines are generally recognized as being highly effective this is the first specific report from Canadian beef herds documenting a beneficial association with use (5).

The survey response rate was slightly lower than the 2016 western Canadian cow-calf survey (88% vs. 81%) and might have been impacted by the severe drought related pressures placed particularly on western producers in 2021 during survey collection (25). Due to the criteria to be enrolled in the study, this group of producers likely represents a progressive subset of producers and might not be reflective of all Canadian cow-calf producers. This cohort of producers represents the early adopters of innovative practices, and are therefore potentially an overrepresentation of vaccine use in the average Canadian cow-calf herd. It is unknown whether responses to the survey or overall vaccination rates were affected by the COVID-19 pandemic or if management decisions were negatively impacted by vaccine hesitancy surrounding viral respiratory vaccines. While surveys suffer from recall bias, producers were provided with color photos and a book to help them find the name of the products used if they did not have access to receipts or records. Misclassification was also managed by asking the producers to report the actual trade name of the product used from their receipts and records rather than asking them what diseases they vaccinated for. The trade names were then re-coded to capture the vaccine components by the study team.

Vaccine use across Canada has shown significant improvement over the past few decades. Cow-calf producers generally followed recommended vaccination protocols for all core vaccines. The number of herds that vaccinated animals increased for nearly all cattle groups since previous reports. One notable change is the increased use of intranasal respiratory vaccines, specifically in neonatal calves. While many positive changes have been made in the industry, there are still some areas where improvement is needed. Producers should be encouraged to vaccinate calves against respiratory disease before 3 months of age, in consultation with their veterinarian, to allow more time for the vaccine to have an effect before high-risk periods for the disease and to allow time to administer a second dose prior to weaning. The literature is scant as to the relative benefits of mucosal vaccination of calves near the time of birth, as well as for the optimal type of follow up vaccination. Recently several projects observing mucosal prime systemic boost have found a benefit for mucosal priming of neonates that is variable depending on the pathogen and form of booster vaccine used; a benefit for mucosal prime and systemic boost has been found for BRSV, bovine coronavirus and BVDV (14–16, 18). Mucosal prime and systemic boost, of neonatal calves, has been shown to improve virus specific and neutralizing antibody concentrations and their booster responses for BRSV, BCoV and BVDV. Improved protection against disease has also been observed when primed and boosted calves were challenged with BRSV or BVDV type 2 (16, 18). More research is needed on the relative benefits of vaccinating calves near time of birth and optimal timing of a booster to minimize risks of respiratory disease associated with stress and increased animal density during activities such as artificial insemination (24, 39, 42).

While all classes of cattle were examined, this study contributes unique information on the types, timing and number of vaccine doses used in nursing calves from a cohort of privately owned herds. While participants likely represent a relatively progressive subset of producers, more producers reported vaccinating nursing calves for BRD as compared to previous work in similar cohorts. The results show that high adoption rates for preweaning vaccination is a reasonable goal for commercial cow-calf herds. However, there was very little consistency in vaccine protocols for nursing beef calves, highlighting opportunities to optimize vaccination timing for challenges specific to each operation such as the timing of calving season and resource limitations. In addition, protocols might vary depending on whether the primary goal of calf vaccination is to reduce disease following weaning, or in nursing calves either before or after summer pasture turnout.

To improve vaccine efficacy, producers must properly follow product labels instructions, specifically regarding administration of booster doses within the specified period. This issue was most apparent for clostridial vaccines in cows and bulls, and scours vaccines in bred heifers. Economic incentives for producers to vaccinate calves prior to feedlot entry should be a top priority for industry to ensure the long-term sustainability of Canadian beef production. There are still a small percentage of cow-calf producers that are not using the core viral vaccines for whom targeted technology transfer activities might be necessary. Finally, clostridial vaccines are underused in cows and bulls, particularly in eastern herds. These vaccines should be administered yearly to ensure cattle are fully protected and optimal clostridial transfer of antibodies.

## Data availability statement

The datasets presented in this article are not readily available because the information was provided by the study participants under the restriction that only summary data would be shared. That is included in the detailed tables and figures provided in this paper. Requests to access the datasets should be directed to cheryl.waldner@usask.ca.

## Ethics statement

The studies involving humans were approved by University of Saskatchewan’s Behavioral Research Ethics Board. The studies were conducted in accordance with the local legislation and institutional requirements. The participants provided their written informed consent to participate in this study.

## Author contributions

CW designed the study and survey tool. CW and ML recoded the data and completed the statistical analyses. ML wrote the manuscript. CW, NE, JC, and SG edited, revised, and provided feedback on the manuscript. All authors contributed to the article and approved the submitted version.

## Funding

Funding provided by Beef Cattle Research Council.

## Acknowledgments

The authors wish to acknowledge the assistance of Sharlene April in managing the survey distribution and data entry for this project. The authors also with to thank the cow-calf producers who participated in the Canadian Cow-Calf Surveillance Network.

## Conflict of interest

The authors declare that the research was conducted in the absence of any commercial or financial relationships that could be construed as a potential conflict of interest.

## Publisher’s note

All claims expressed in this article are solely those of the authors and do not necessarily represent those of their affiliated organizations, or those of the publisher, the editors and the reviewers. Any product that may be evaluated in this article, or claim that may be made by its manufacturer, is not guaranteed or endorsed by the publisher.
